# Childhood socioeconomic status and childhood maltreatment: Distinct associations with brain structure

**DOI:** 10.1371/journal.pone.0175690

**Published:** 2017-04-17

**Authors:** Gwendolyn M. Lawson, Joshua S. Camins, Laura Wisse, Jue Wu, Jeffrey T. Duda, Philip A. Cook, James C. Gee, Martha J. Farah

**Affiliations:** 1 Psychology Department, University of Pennsylvania, Philadelphia, Pennsylvania, United States of America; 2 Penn Image Computing & Science Lab, University of Pennsylvania, Philadelphia, Pennsylvania, United States of America; Central Institute of Mental Health, GERMANY

## Abstract

The present study examined the relationship between childhood socioeconomic status (SES), childhood maltreatment, and the volumes of the hippocampus and amygdala between the ages of 25 and 36 years. Previous work has linked both low SES and maltreatment with reduced hippocampal volume in childhood, an effect attributed to childhood stress. In 46 adult subjects, only childhood maltreatment, and not childhood SES, predicted hippocampal volume in regression analyses, with greater maltreatment associated with lower volume. Neither factor was related to amygdala volume. When current SES and recent interpersonal stressful events were also considered, recent interpersonal stressful events predicted smaller hippocampal volumes over and above childhood maltreatment. Finally, exploratory analyses revealed a significant sex by childhood SES interaction, with women’s childhood SES showing a significantly more positive relation (less negative) with hippocampus volume than men’s. The overall effect of childhood maltreatment but not SES, and the sex-specific effect of childhood SES, indicate that different forms of stressful childhood adversity affect brain development differently.

## Introduction

Childhood poverty and maltreatment both have lasting effects on cognitive development and mental health. Although the two forms of adversity differ from one another [1], both have been explained by the effects of stress on the developing brain. In the case of maltreatment, both neglect and abuse would be expected to increase children’s stress. In the case of poverty, insecurity related to food, shelter, safety and other concomitants of low socioeconomic status would also increase stress. However, the experiences associated with childhood poverty and maltreatment differ in many ways, including the threat of harm, frequency of exposure, and chronicity [2], and it may not be appropriate to assume that both sets of experiences affect the developing brain through the same mechanisms.

Growing literatures examine the structural correlates of childhood maltreatment during childhood and in adulthood (e.g., [3–6]). Similarly, the correlates of childhood poverty have been studied in the child and adult brain (e.g., [7–10]**)**. However, there is a dearth of studies directly comparing childhood maltreatment and childhood socioeconomic status (SES). Although there is ample evidence for the involvement of stress in both, more direct comparisons are needed to determine the extent to which these experiences affect brain development through similar or distinct pathways. The present study examines the association between childhood SES, childhood maltreatment and hippocampal and amygdala volume in early adulthood in order to examine the similar or distinct correlates of childhood SES and maltreatment.

The most-studied aspect of brain structure in childhood adversity is the hippocampus, which is sensitive to stress experiences as well as playing a role in the regulation of the stress response. Its neighbor in the medial temporal lobe, the amygdala, has also been found to correlate with childhood maltreatment and poverty in some studies. We focus the present investigation on the volumes of these structures in early adulthood.

Concerning childhood maltreatment and the structural development of the hippocampus, a recent meta-analysis found that, across 49 studies of children and adults, experiences of maltreatment were associated with significantly reduced hippocampal volume [11]. However, evidence of reduced hippocampal volume following childhood maltreatment is more consistent in adulthood (e.g., [5]) than during childhood (e.g., [12,13]). Indeed, in the aforementioned meta-analysis, when studies of children and adults were examined separately, the overall effect size for studies of adults was significant, but the effect size for studies of children was not [11].

More recently, researchers have started to examine the relationship between childhood SES and hippocampal volume. The literature is remarkably consistent, showing smaller hippocampi in children living in lower SES environments [3,7–9,14,15], a finding that has been interpreted in terms of the child’s experienced stress (see [8] for particularly direct evidence for this interpretation).

It is not clear, however, the extent to which these differences persist into adulthood. In a study of middle-aged adults, childhood poverty was unrelated to hippocampal volume, although financial hardship in adulthood did relate to smaller hippocampal volume [16]. However, another study observed a positive association between childhood SES and hippocampal volume in late adulthood [10]. Consistent with the idea that SES differences in hippocampal volume may re-emerge in later adulthood, Noble et al. (2012) found that education in adulthood moderated age-related decreases in hippocampal volume, such that differences in hippocampal volume associated with education were observed in older, but not younger, adults [17].

For the amygdala, findings on effect of childhood maltreatment are less consistent. Studies have reported larger amygdalae in children who experienced early institutional deprivation, which could be considered a form of child neglect [18,19] and in adults with exposure to childhood maltreatment [4]. Other reports have noted smaller amygdala in maltreated children [3,20], and still others have reported no differences in amygdala volume associated with childhood maltreatment [12,13,21].

Similarly, findings on childhood SES and amygdala volume are inconsistent. Published studies have found no significant relationship between SES and amygdala volume [9,14], a negative correlation such that higher SES is associated with a smaller amygdala [15], and a positive correlation such that higher SES is associated with a larger amygdala [3,8].

While the neurobiological correlates of maltreatment and poverty have largely been considered separately, a recent study bridges these literatures by considering the structural correlates of both experiences, conceptualized as different forms of early life stress [3]. This study compared hippocampal and amygdala volumes among four groups: children who experienced early neglect, children who experienced physical abuse, children from low-SES households, and children who experienced none of these adversities. The three early life stress groups showed qualitatively similar reductions in the volume of the left and right hippocampus and the left amygdala, compared to the comparison sample. Further, for children exposed to any form of early life stress, higher levels of cumulative life stress predicted smaller volumes of the left amygdala and the hippocampus. These results suggest similar mechanisms are at work among these different forms of early life stress [3].

The present study took a different approach to the question of whether childhood maltreatment and low SES affect the brain by common or distinct mechanisms. We studied the neural correlates of childhood SES and childhood maltreatment in a single sample. Childhood SES and childhood maltreatment tend to be correlated in the general population (e.g., [22], but are often examined separately, without controlling for the other. As such, the separate contribution of each construct to differences in brain structure, and the extent to which these factors operate similarly or differently, is not yet clear. Although we do not directly measure or manipulate the potentially mediating processes giving rise to the effects of childhood adversity on the brain, we reason that if the effects are different then the processes that gave rise to them must also have been different.

To assess the lasting consequences of these potentially distinct effects, we measured childhood maltreatment and childhood SES in young adult participants. To assess the extent to which these effects are themselves distinct from similar experiences in adulthood, which would also be expected to influence the adult brain, we conducted additional analyses including measures of recent SES and interpersonal stressors. For the sake of thoroughly examining the relation between childhood SES and adult hippocampal volume, we carried out exploratory tests of the moderation of that relation by sex.

We employed structural MRI data from a socioeconomically diverse sample of young adults, an age range that has been understudied regarding childhood SES and brain structure. Based on the extensive literatures on stress and the hippocampus and amygdala, we focus on the volumes of these limbic structures and examine the similar or distinct correlates of childhood SES and childhood maltreatment.

## Materials and methods

### Participants

This study was approved by the University of Pennsylvania IRB #7, Protocol Number: 819312. The sample included N = 46 young adults (50% male) between the ages of 25 and 36 (mean age = 28.15; SD = 2.76) recruited from the Philadelphia area and provided written consent. Participants were recruited through advertisements on Facebook, Craigslist and flyers placed in public places around Philadelphia. The sample was intentionally recruited to have a wide range of current educational levels, from less than high school to graduate degrees. To avoid racial confounds, the sample was limited to participants who self-reported their race Caucasian. 44 participants identified as non-Hispanic Caucasian, and 2 participants (both male) identified as Hispanic Caucasian.

Individuals were also excluded from participation if they were pregnant, had a body mass index over 40, reported contraindications to MRI scanning, had a history of any neurological disorder, experienced a traumatic brain injury or concussion with loss of consciousness, had ever received a diagnosis of bipolar disorder or any psychotic disorder or had ever taken an antipsychotic medication. Participants were also removed if they indicated excess drinking for more than 6 months (3 drinks per day for men, 2 drinks per day for women) or use of any drug other than cannabis more than 6 times.

We aimed to recruit a sample size of 60, based on the sample sizes of other studies with substantial proportions of low SES participants (61 in the case of [3] and 23 in the case of [7]). We stopped data collection at the termination of the grant, at which time 48 were collected.

Two subjects who completed the scan were excluded from the sample. In one case, an incidental finding that required medical follow-up was discovered. In the second case, the participant’s behavior was erratic (e.g., illogical and incoherent speech) and elicited concern from the MRI technician. Results were similar when these subjects were included.

### Measures

#### Childhood SES

Three components of childhood socioeconomic status were measured: parental education, parental occupational prestige, and childhood financial circumstances. These variables were z-standardized and averaged to create a childhood SES composite measure.

For *parental education* participants reported on the educational attainment of their parents/guardians at the time they were born. Each parent’s education level was assigned a value from 1 to 6 (Less than High School = 1, High School = 2, General Education Diploma (GED) = 3, Some College/Associates Degree = 4, 4-year College Degree = 5, Graduate Level = 6). Educational levels for the first and second parent/guardian were z-standardized and were averaged to compute the parental education variable. When a participant reported that there was no second parent/guardian, the *z*-standardized education level of the first parent/guardian was used. For 44 subjects (95.7%) the first parent/guardian was a mother. For 36 subjects (78.3%) the second parent/guardian was a father.

For *parental occupation*, participants described each parent/guardians occupation during the first 5 years of the child’s life in a semi-structured interview. Occupations were scored using the Hollingshead index [23]. Occupational prestige scores for the first and second parent/guardian were z-standardized and were averaged to compute the parental occupation variable.

For c*hildhood financial circumstances*, five questions were administered. Three questions (“My family usually had enough money for things when I was growing up,” “I grew up in a relatively wealthy neighborhood,” and “I felt relatively wealthy compared to other kids in my school”) were answered on a 7-point Likert scale [24]. Two questions (“When you were a child, was your father or mother unemployed when they wanted to be working?,” “When you were a child, did your family have continuing financial problems?”) were answered with yes/no [25]. Scores for each question were z-standardized and reverse scored as appropriate such that higher scores indicate higher levels of childhood financial security and the five questions were averaged together to compute the childhood financial circumstances variable. The scale had good internal consistency (α = .81). An additional yes/no question (“When you were a child, did your family have a car?”) was included in the questionnaire but removed from the final scale because of a low item-total correlation (*r* = .25).

#### Childhood maltreatment

Participants completed the Adverse Childhood Experiences (ACE) [26] questionnaire, which asks individuals to indicate whether or not they experienced each of ten possible adverse events as a child. Not all of these adverse events constitute maltreatment. Therefore, a subset of the ACE questions was used to measure childhood maltreatment, specifically the following six items, which assess childhood abuse, neglect, or exposure to domestic violence, with their original ACE questionnaire numbering: 1. Did a parent or other adult in the household often or very often swear at you, insult you, put you down, or humiliate you or act in a way that made you afraid that you might be physically hurt? 2. Did a parent or other adult in the household often or very often push, grab, slap, or throw something at you or ever hit you so hard that you had marks or were injured? 3. Did an adult or person at least 5 years older than you ever touch or fondle you or have you touch their body in a sexual way or try to or actually have oral, anal, or vaginal sex with you? 4. Did you often feel that no one in your family loved you or thought you were important or special or your family didn’t look out for each other, feel close to each other, or support each other? 5. Did you often or very often feel that you didn’t have enough to eat, had to wear dirty clothes, and had no one to protect you or your parents were too drunk or high to take care of you or take you to the doctor if you needed it? 7. Was your mother or stepmother often or very often pushed, grabbed, slapped, or had something thrown at her, or sometimes, often, or very often kicked, bitten, hit with a fist, or hit with something hard or ever repeatedly hit at least a few minutes or threatened with a gun or knife? Given the preponderance of low scores in our sample and community samples more generally, this variable was square root transformed for use in analyses.

#### Current SES

Two components of current socioeconomic status were measured: current educational attainment and current financial security. These variables were z-standardized and averaged to create a childhood SES composite measure. Current SES was measured in as similar of a way to childhood SES as possible, given that the construct of SES differs between childhood and adulthood.

For *current education*, participants reported on their current educational attainment. Each individual’s education level was assigned a value from 1 to 6 (Less than High School = 1, High School = 2, General Education Diploma (GED) = 3, Some College/Associates Degree = 4, 4-year College Degree = 5, Graduate Level = 6).

For *financial security*, participants completed a questionnaire assessing their current level of financial strain. Six questions, all of which indicated current difficulty affording necessities and have been used in prior studies of financial strain, were used. Five questions (“How hard is it for you and your family to pay for the basics like food, medical care, and heating?” “How well does your income cover your needs?” “How difficult have you found paying bills lately?” “In the past two years, how often have you decided not to buy something you or your family needed because you couldn’t afford it?” and “In the past two years, how often have you borrowed money from family or friends to pay bills or to make ends meet?”) were answered on four-point Likert scales [27–29]. Scores for each question were z-standardized and reverse scored as appropriate such that higher scores indicate higher levels of financial security; the six questions were then averaged together to compute the current financial security variable. The scale had excellent internal consistency (α = .97).

#### Recent negative interpersonal events

Participants completed a modified version of the Life Events Questionnaire [30]. This measure provided a list of 44 major life events (e.g., death of a close family member, major personal illness or injury); participants were instructed to indicate whether each event had occurred to them in the past year, and, if so, to rate the impact it had on their lives (on a 7-point Likert scale from extremely negative to extremely positive). We obtained a negative events score by summing the impact rating for those events rated as having a negative impact by the subject. Additionally, we created a negative interpersonal events score by calculating the negative events score for the subsample of 21 events that are inherently interpersonal in nature. This score was used in analyses in order to use a measure that is as comparable as possible to childhood maltreatment, which is inherently interpersonal in nature. Results were similar when the total negative events score was used.

#### Covariates

Four variables that might reasonably be expected to correlate with hippocampal or amygdala volume include age, sex, BMI and total brain volume. The inclusion of total brain volume as a control variable allows us to examine specific associations between the factors examined and our regions of interest, above and beyond any more global effects. To assess the effects of childhood maltreatment and childhood SES independent of these factors, they served as covariates in the analyses to be reported. In secondary analyses to be reported, the interaction of sex with maltreatment and SES is also considered.

### Image processing

All images were acquired on a Siemens Trio 3.0 Tesla MRI scanner. At the start of each scanning session, patient position was determined using a rapid coronal T1-weighted scan. This was followed by a T1-weighted structural scan with TR (repetition time) = 1810 ms, TE (echo time) = 3.51 ms, slice thickness: 1 mm, in-plane resolution: 0.9375 x 0.9375 mm and field of view (FOV) 192 x 256 x 160 mm.

The T1 imaging data were preprocessed using the open-source Advanced Normalization Tools (ANTs; [31]). The provided antsCorticalThickess.sh script performed automated brain extraction as well as inhomogeneity correction [32]. The right and left hippocampus was segmented using multi-atlas label fusion with error correction [33], implemented as the AHEAD tool (https://www.nitrc.org/projects/ahead/). AHEAD includes a library of manually segmented hippocampi to label individual subjects via image registration and joint label fusion. The error correction is specialized for hippocampus only. For the amygdala segmentation, we used a general label fusion algorithm implemented ANTs. The atlases for this procedure were 24 healthy adults from the OASIS project [34], segmented manually by Neuromorphometrics, Inc. (http://Neuromorphometrics.com/) and provided under academic subscription as part of a segmentation workshop (https://masi.vuse.vanderbilt.edu/workshop2012/index.php/Main_Page). We selected the youngest 24 of the 30 available atlases, to better match the age of the subjects in this study. The subset consists of 16 females and 8 males aged 18–45, mean age 25.

The automated segmentations were reviewed and corrected manually by LW. This resulted in edits to hippocampus segmentations for 7 individuals and amygdala segmentations for 5 individuals. The median volume change after editing was 2% for both structures.

### Statistical approach

Analyses used hierarchical linear regression to predict volume in each region of interest. Two-tailed *p* values are reported. Control variables (age, sex, BMI, total brain volume) were entered in Step 1. In Step 2, Childhood SES and childhood maltreatment were then added. To detect collinearity between childhood SES and childhood maltreatment, which could distort the results of the multiple regressions reported here, we verified that they were not strongly correlated and also confirmed that the regression results for each were similar when entered separately (in separate steps) and together.

Next, to examine the specific importance of *childhood* SES and maltreatment, independent from current SES and recent interpersonal stress, we repeated the step 2 models also including current SES and recent negative interpersonal events as current covariates. To examine the possibility that maltreatment exacerbates the effect of recent stress on the hippocampus and amygdala (e.g., consistent with the stress sensitization model; [35]), we also ran exploratory models including an interaction between recent negative interpersonal events and childhood maltreatment.

Finally, in Step 3, interaction terms between the variables of interest and sex were added. When a significant interaction was identified, regression models were run separately for each sex group.

## Results

### Participant characteristics

The sample was diverse in terms of both childhood SES and maltreatment exposure. Not surprisingly, given that participants were recruited to have widely varying adult SES as measured by educational attainment, the childhood SES of these participants also varied widely. The mean Hollingshead occupation score for the first parent/guardian was 5.44 (SD = 1.86; range: 1 to 8) and the mean Hollingshead occupation score for the second parent/guardian was 5.63 (SD = 2.51; range 1 to 9). 45.7% of first parent/guardians and 39.1% of second parent/guardians did not have educational attainment beyond a high school degree. Childhood maltreatment, abuse and exposure to domestic violence also varied in this sample. 37% of the sample endorsed one or more items from the 6-item abridged ACE questionnaire, with the remaining 63% endorsing none. The preponderance of no endorsements is expected based on findings from US community samples. For example, 46% of US respondents endorsed one or more items, 54% of respondents endorse none, and the two most commonly reported adverse experiences were economic hardship and divorce [36]. These were excluded from the abridged version used here, predicting a higher proportion of responses endorsing none. Descriptive statistics are displayed in Table 1.

**Table 1:** Descriptive statistics of caregiver education and childhood maltreatment scores for analytic sample (n = 46).

Variable	n (%)
First parent/guardian education	
Less than High School	3 (6.5)
GED	1 (2.2)
High School Graduate	17 (37.0)
Some College or Associates Degree	7 (15.2)
4-Year College Degree	17 (37.0)
Graduate Degree	1 (2.2)
Second parent/guardian education	
Less than High School	2 (4.3)
GED	3 (6.5)
High School Graduate	13 (28.3)
Some College or Associates Degree	6 (13.0)
4-Year College Degree	14 (30.4)
Graduate Degree	4 (8.7)
No secondary caregiver or unknown	4 (8.7)
Maltreatment score	
0	29 (63.0)
1	6 (13.0)
2	5 (10.9)
3 or greater	6 (13.0)

*Note*. GED = General Education Diploma

#### Correlations

Correlations between the variables of interest and covariates are displayed in Table 2. Note that childhood SES and childhood maltreatment, whose distinctive effects we are examining, are only weakly correlated (r = -.28, *p* = .06).

**Table 2:** Correlation matrix.

Indicator	1	2	3	4	5	6	7	8	9	10	11	12
1. Childhood SES	1											
2. Childhood maltreatment	-.28+	1										
3. Current SES	.27+	-.36*	1									
4. Recent negative interpersonal events	.11	-.22	.53**	1								
5. Male	.35*	-.18	-.24	-.15	1							
6. Age	.05	.33*	-.28+	-.07	.17	1						
7. Body mass index	-.12	.19	-.06	.13	.01	.42**	1					
8. Brain volume	.24	-.12	-.11	.09	.60**	.03	-.05	1				
9. Volume of the left hippocampus	.23	-.30*	.03	-.03	.48**	.09	.07	.68**	1			
10. Volume of the right hippocampus	.31*	-.29+	.04	-.03	.51**	.04	.03	.77**	.89**	1		
11. Volume of the left amygdala	.17	-.16	-.14	.06	.52**	.04	.07	.74**	.68**	.70**	1	
12. Volume of the right amygdala	.16	-.18	-.23	.09	.66**	.07	-.06	.73**	.55**	.63**	.88**	1

Correlation matrix presenting correlations for childhood and current SES, childhood maltreatment, recent negative interpersonal events, regional brain volumes, and covariates

*Note*. SES = socioeconomic status.

**p < .01

*p < .05

+*p* < .1

### Regression results

#### Main effects of childhood SES and childhood maltreatment

For the analysis of hippocampal volume, when childhood SES was added to the model along with covariates (age, sex, BMI, total brain volume), childhood SES was not predictive for the left hippocampus (β = .06, *p* = .62) or right hippocampus (β = .14, *p* = .21). In the model with childhood maltreatment and covariates, higher levels of childhood maltreatment significantly predicted smaller volumes of the left hippocampus (β = -.27, *p* = .03) and the right hippocampus (β = -.24, *p* = .03). Similarly, when childhood SES and childhood maltreatment were added to the model simultaneously, childhood SES did not significantly relate to the volume of the left or right hippocampus, but higher levels of childhood maltreatment significantly predicted smaller volume of the left hippocampus (β = -.28, *p* = .03) and the right hippocampus (β = -.22, *p* = .048). These results are shown in Table 3.

**Table 3:** Results from hierarchical multiple linear regression using control variables (age, sex, BMI, total brain volume), followed by childhood SES and childhood maltreatment to predict volume of the hippocampus and amygdala.

	Region of interest							
	Left hippocampus		Right hippocampus		Left amygdala		Right amygdala	
Predictor	Δ R^2^	β	Δ R^2^	β	Δ R^2^	β	Δ R^2^	β
Step 1	.48**		.60**		.57**		.61**	
Control variables								
Step 2	.06+		.05+		.01		.02	
Childhood SES		-.01		.08		-.05		-.13
Childhood maltreatment		-.28*		-.22*		-.09		-.10
Total R^2^	.54**		.65**		.58**		.63**	
*n*	46		46		46		46	

*Note*. SES = Socioeconomic Status.

** p < .01

*p < .05

+ p < .1

In the analysis of amygdala volume, neither childhood SES nor childhood maltreatment was predictive, either alone or in the fully adjusted model. These results are shown in Table 3.

#### Effects of current SES and recent interpersonal stress exposure

**Fig 1:**
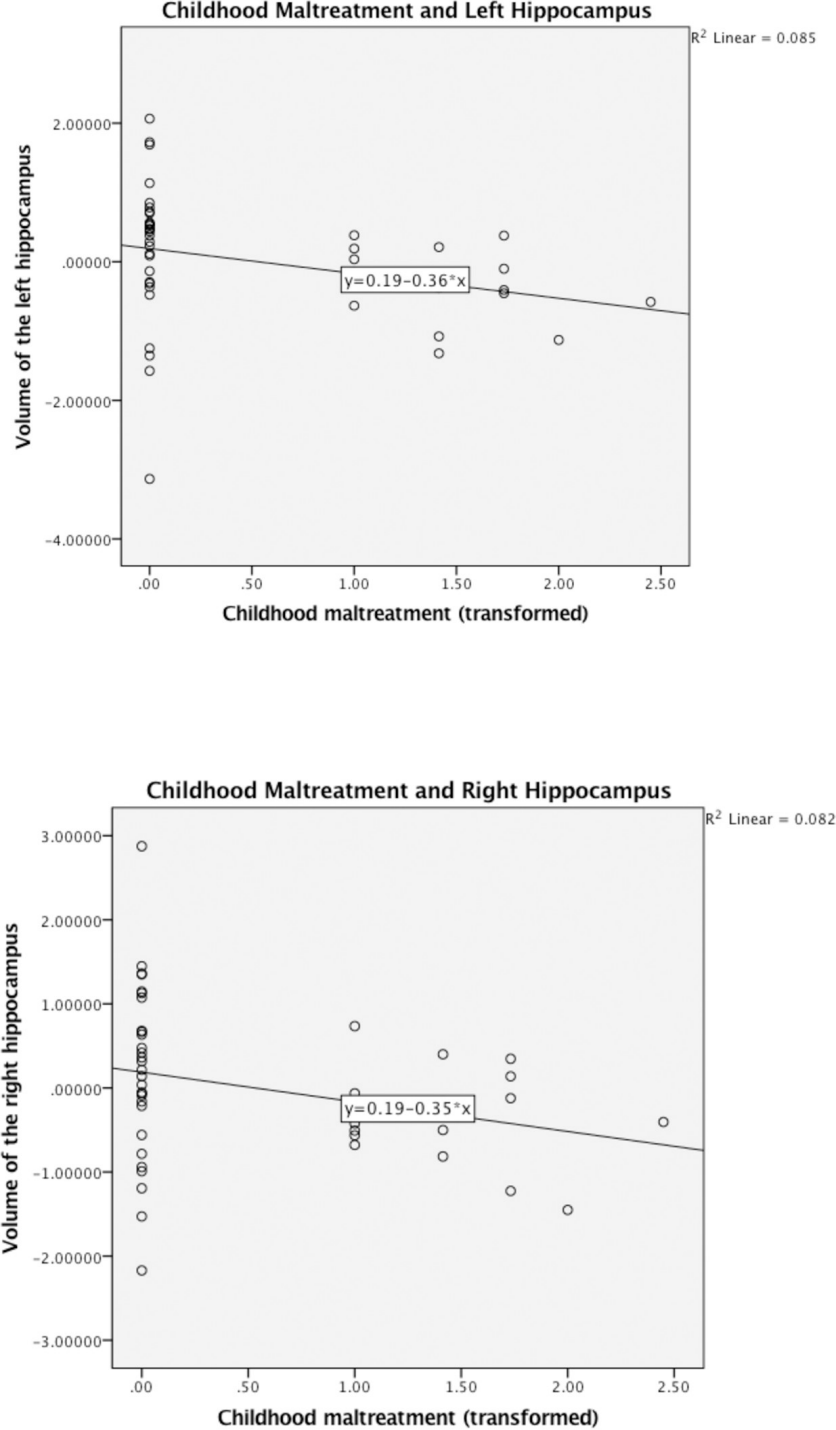
Hippocampal volume as a function of childhood maltreatment, adjusted for childhood SES, current SES, recent negative interpersonal events, sex, age, total brain volume, and BMI.

Table 4 shows the results of analyses in which current SES and recent negative interpersonal events were added to the model along with childhood SES and childhood maltreatment and covariates. Recent negative interpersonal events had a significant negative relationship with the volume of the right hippocampus (β = -.29, *p* = .02) and a marginally significant negative relationship with the volume of the left hippocampus (β = -.26, *p* = .06). Current SES was not significantly associated with left (β = .19, *p* = .19) or right (β = .19, *p* = .13) hippocampal volume. Childhood maltreatment remained a significant negative predictor of hippocampal volume for the left and right hippocampus when these covariates were included in the model. Scatterplots of the relation between childhood maltreatment and left and right hippocampal volume are shown in Fig 1.

**Table 4:** Results from hierarchical multiple linear regression using control variables (age, sex, BMI, total brain volume), variables of interest (childhood SES, childhood maltreatment, current SES, and recent negative interpersonal events), and interactions of these variables with sex, to predict volume of the hippocampus and amygdala.

	Region of interest							
	Left hippocampus		Right hippocampus		Left amygdala		Right amygdala	
Predictor	Δ R^2^	β	Δ R^2^	β	Δ R^2^	β	Δ R^2^	β
Step 1	.48**		.59**		.57**		.61**	
Control variables								
Step 2	.11+		.11*		.01		.05	
Childhood SES		-.04		.06		-.02		-.09
Childhood maltreatment		-.29*		-.24*		-.12		-.11
Current SES		.19		.20		-.10		-.22
Recent negative interpersonal events		-.26+		-.29*		.02		.21+
Step 3	.10+		.05		.05		.06	
Sex x Childhood SES		-.42*		-.32*		-.15		-.03
Sex x Childhood maltreatment		-.05		-.04		.23		.30*
Sex x Current SES		-.10		.25		-.15		.41*
Sex x Recent negative interpersonal events		.52		-.21		-.04		-.38
Total R^2^	.68**		.75**		.77**		.73**	
*n*	46		46		46		46	

*Note*. SES = Socioeconomic Status.

** *p* < .01

*p < .05

+ *p* < .1

We also examined the interaction between recent negative interpersonal events and childhood maltreatment, motivated by the stress sensitization model of Hammen et al. [35]. The interaction term did not significantly predict the volume of the left (β = .20; p = .59) or the right (β = -.09; p = .78) hippocampus.

In analyses of amygdala volume using the same covariates, recent negative interpersonal events had a marginally significant positive relationship with the volume of the right amygdala (β = .21, *p* = .08) and did not predict the volume of the left amygdala (β = .02, *p* = .18). Current SES did not predict the volume of the right (β = -.22, *p* = .10) or left (β = -.10, *p* = .48) amygdala. These results are shown in Table 4.

We also examined the interaction between recent negative interpersonal events and childhood maltreatment. This interaction term did not significantly predict volume of the amygdala.

Given the robust evidence for SES effects on hippocampal volume in childhood, we were somewhat surprised by the absence of a childhood SES effect on the hippocampus in the present data. To more thoroughly assess this relationship we examined it as a function of sex in exploratory analyses.

We added interaction terms for sex with each of the four variables of interest (childhood SES, childhood maltreatment, current SES, recent negative interpersonal events). The interaction between sex and childhood SES was significant for the model predicting left hippocampal (β = -.42, *p* = .01) and right hippocampal (β = -.32, *p* = .03) volume. The interaction terms between sex and childhood maltreatment, current SES, and recent negative interpersonal events were not significant. These results are shown in Table 4.

**Fig 2:**
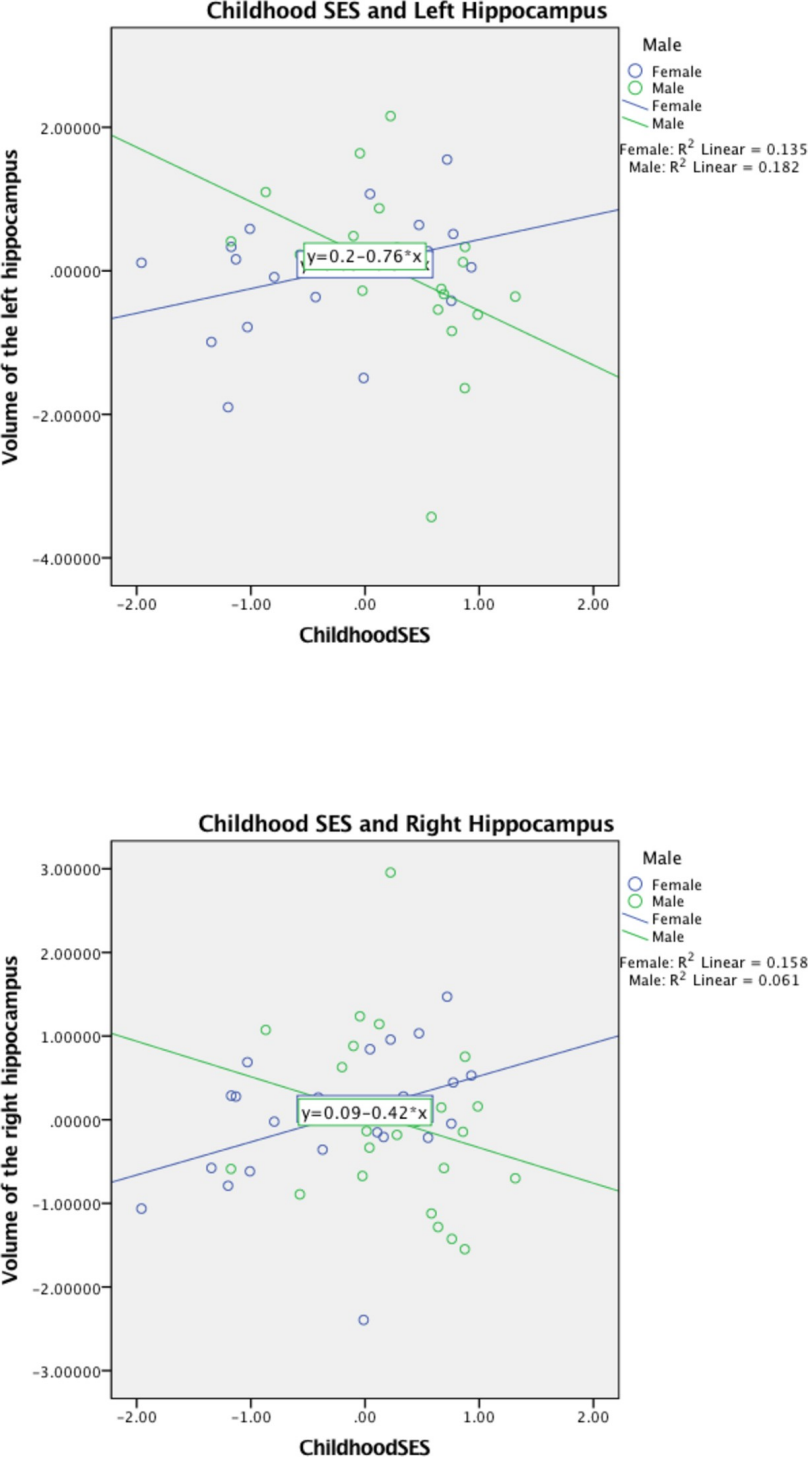
Hippocampal volume as a function of childhood SES for males and females., adjusted for childhood maltreatment, current SES, recent negative interpersonal events, age, total brain volume, and BMI.

Sex subgroups were then examined separately. In the female subgroup, childhood SES was not significantly related to left (β = .25, *p* = .20) or right (β = .27, *p* = .11) hippocampal volumes. In the male subgroup, childhood SES had a marginally significant negative relationship to left hippocampal volume (β = -.43, *p* = .08) and no significant relationship to right hippocampal volume (β = -.32, *p* = .15). Scatterplots of the relation between childhood SES and left and right hippocampal volume, split by sex group, are shown in Fig 2.

For completeness we used the same model, with sex interactions, to predict amygdala volume in a further exploratory analysis. As shown in Table 4, both the interaction between sex and current SES (β = .42, *p* = .04) and the interaction between sex and childhood maltreatment (β = -.30, *p* = .03) were significant for the model predicting the right amygdala. None of the interaction terms significantly predicted the volume of the left amygdala. In each case with significant interaction, the pattern was that higher adversity associated with higher right amygdala volume in females and with lower right amygdala volume in males. This trend was significant for current SES in females (β = -.48, *p* = .03) but not for childhood maltreatment (β = -.31, *p* = .14), and was not significant in males (β = .21, *p* = .43 and β = .42, *p* = .14 for current SES and childhood maltreatment, respectively).

## Discussion

Low SES and maltreatment are associated with decreased hippocampal volume in children, a parallel that has been attributed to the role of stress in both. In the present study we found evidence that these well-established effects of childhood experience diverge in adulthood. Only childhood maltreatment showed a main effect on hippocampal volume and only childhood SES showed an interaction with sex. These differing patterns of effect, in the same sample of participants, suggest that the two forms of childhood adversity affect the brain through distinctive mechanisms. This may reflect differences in the specific nature of a child’s experiences in the context of maltreatment and poverty. The intensity and duration of threat exposure, the fate of attachment processes (which may affect stress physiology, [37]) and levels of cognitive stimulation seem likely to differ, on average, between maltreatment and poverty [2]. Future research should examine potential mechanisms by measuring these proximal factors, in addition to childhood SES and maltreatment, ideally in the same sample of participants.

In addition to childhood maltreatment, recent interpersonal stress in adulthood was also associated with smaller hippocampal volumes. Most evidence linking adulthood stress to hippocampal volume comes from samples with stress-related psychopathology (e.g., [38]), unlike the present community sample. The other such study, by Gianaros and colleagues (2007), found that chronic life stress was associated with decreased grey matter volume in the right hippocampus in a sample of healthy postmenopausal women [39]. The current results extend these findings by showing an association between recent stressful life events and hippocampal volume (again on the right, although borderline significant on the left as well) in a sample of healthy young men and women.

These results also speak to important questions about the impact of the timing of adverse experiences. Does childhood adversity, independently from adversity in adulthood, shape adulthood outcomes? Is childhood a period of particular vulnerability to adversity? Does childhood adversity potentiate the impact of adulthood stress? While the current study is not designed to answer these questions conclusively, the results are most consistent with a model in which childhood and adulthood stress independently shape brain structure in early adulthood. Indeed, we observed significant main effects of childhood maltreatment and recent stress, but not an interaction between these factors.

The results regarding interactions with sex emerged from exploratory analyses and were not obtained with specific a priori hypotheses in mind. They are reported here as a modest empirical contribution to the literature on early life stress, adult neuroanatomical differences and sex. We cannot interpret them with any confidence at the present, although the finding that childhood SES and sex interact to predict hippocampal volume could be another manifestation of sex differences in the development of stress regulation systems and the associated neurobiology [40, 41].

Regarding the amygdala, the absence of findings here is not unexpected given the inconsistences in the literature reviewed earlier. The reason for the differences between hippocampal and amygdalar volume effects may be related to the different functions of the two structures, their different sensitivities to stress at different points in development and differences in the developmental processes that manifest in overall volume are all possible reasons. An additional possibility may relate to the difficulty of segmenting the amygdala. Hanson et al. (2014) found that manually traced amygdala volumes revealed associations with early life stress whereas automatically segmented amygdala volumes did not, raising the possibility that other null results are attributable to imprecise segmentation [3]. Although we did not manually segment all amygdalas in the present study, we did check the automatic segmentations visually and then manually corrected segmentations as needed. We nevertheless failed to find main effects of adversity on amygdala volume.

The clinical implications of the present findings have less to do with hippocampal structure per se than with the more general caution they raise against assuming the equivalence of different forms of childhood adversity, even those for which abnormal levels of stress play a crucial role. These findings remind us that clinically relevant vulnerabilities, therapies and outcomes discovered for one form of early, stress-linked adversity may not necessarily generalize to the other.

There are a number of limitations to the current study. First, the study is limited by its relatively small sample size and the associated limited power. This is a particular concern when interpreting null results (e.g., the lack of main effect observed between childhood SES and hippocampal volume). However, it is important to note that our SES variation was maximized by recruiting equal proportions of subjects in categories from high school dropout to graduate degree, and in the same model childhood maltreatment did relate to hippocampal volume.

A second limitation is that the measures used for childhood SES and childhood maltreatment differed from the measures used for adulthood SES and adulthood interpersonal stressors. As such, it was not possible to conclusively separate the timing of SES and interpersonal stress experiences from the measurement approach. However, this is not simply a measurement limitation; the experiences related to SES and maltreatment/interpersonal stress are inherently distinct between childhood and adulthood. As such, it is appropriate to measure these constructs differently. Similarly, it is possible that childhood maltreatment may have been measured with more reliability than childhood SES. Importantly, however, more items were used in the measurement of childhood SES than childhood maltreatment. The current study used data from a single time point, a limitation that precludes strong conclusions about the developmental trajectory of brain development in relation to childhood and adulthood adversity. Despite these limitations, the current study advances the literature on early life stress and brain development by measuring childhood maltreatment and childhood SES within the same young adult sample, which encompassed an unusually wide SES range. The different effects on adult hippocampal volume suggest that childhood maltreatment and childhood SES likely impact the brain through distinct pathways.
